# Classification and Prediction of Skyrmion Material Based on Machine Learning

**DOI:** 10.34133/research.0082

**Published:** 2023-03-15

**Authors:** Dan Liu, Zhixin Liu, JinE Zhang, Yinong Yin, Jianfeng Xi, Lichen Wang, JieFu Xiong, Ming Zhang, Tongyun Zhao, Jiaying Jin, Fengxia Hu, Jirong Sun, Jun Shen, Baogen Shen

**Affiliations:** ^1^ Department of Physics, School of Artificial Intelligence, Beijing Technology and Business University, Beijing 100048, P. R. China.; ^2^ School of Integrated Circuit Science and Engineering, Beihang University, Beijing 100191, China.; ^3^ Ningbo Institute of Materials, Technology & Engineering, Chinese Academy of Sciences, Zhejiang 315201, P. R. China.; ^4^ School of Physics, Inner Mongolia University of Science and Technology, Baotou 014010, P. R. China.; ^5^ State Key Laboratory of Magnetism, Institute of Physics, Chinese Academy of Sciences, Beijing 100190, P. R. China.; ^6^ School of Materials Science and Engineering, Zhejiang University, Hangzhou 310027, P. R. China.; ^7^ Key Laboratory of Cryogenics, Technical Institute of Physics and Chemistry, Chinese Academy of Sciences, Beijing 100190, P. R. China.

## Abstract

The discovery and study of skyrmion materials play an important role in basic frontier physics research and future information technology. The database of 196 materials, including 64 skyrmions, was established and predicted based on machine learning. A variety of intrinsic features are classified to optimize the model, and more than a dozen methods had been used to estimate the existence of skyrmion in magnetic materials, such as support vector machines, *k*-nearest neighbor, and ensembles of trees. It is found that magnetic materials can be more accurately divided into skyrmion and non-skyrmion classes by using the classification of electronic layer. Note that the rare earths are the key elements affecting the production of skyrmion. The accuracy and reliability of random undersampling bagged trees were 87.5% and 0.89, respectively, which have the potential to build a reliable machine learning model from small data. The existence of skyrmions in LaBaMnO is predicted by the trained model and verified by micromagnetic theory and experiments.

## Introduction

Magnetic skyrmions are expected to be the next generation of information carriers with high capacity, high-speed reading and writing, low-power consumption, and nonvolatile information storage [1,2]. The skyrmions observed in the experiments are smaller than typical domain wall lengths, and the topological protection contributes to superior thermal stability, showing the exciting potential for encoding vast amounts of information [3,4]. The current density required to move skyrmion is about 10^6^ A/m^2^, which is 4 to 5 orders of magnitude smaller than moving ferroelectric domain walls [4–6]. Due to the advantages of nanoscale, topological protection, and low spin current regulation, skyrmions are considered promising for spintronic applications. It is feasible to study skyrmions by traditional experimental methods of preparation and observation, but the randomness of the results and the variety of costs are unaffordable for ordinary institutions [7–10]. The essential reason for the low throughput of experimental data generation is the time-consuming and expensive synthesis process. Moreover, microscopic characterization is also required to reliably report the crystal structure and magnetism of these alloys, which adds to the difficulty of research [11,12].

To date, more and more attention has been paid to the application of computational methods in alloy design. Based on first-principles calculations with multiple scattering Korringa–Kohn–Rostoker formalism, Mankovsky et al. [13] demonstrated that the strength of Dzyaloshinskii–Moriya (DM) term was strongly dependent on alloy substitution and epitaxial strain. Density functional theory (DFT) calculations have also been applied to study the electron dynamics in the skyrmion phase of Fe-rich Mn_1−*x*_Fe*_x_*Ge alloys [14]. However, computational efforts involving DFT have mainly focused on interpreting or extracting properties from the perspective of band theory [14–18]. In fact, the principles of alloy theory and the periodic table offer more avenues for composition design than what has been explored in the literature. For example, Shimono et al. [19] developed the statistical method based on machine learning to predict the chemical categories ideally suitable for creating chiral molecules. This model employs a probabilistic classifier and an artificial neural network, which further enhances the deep learning approach of previous work.

Artificial intelligence combined with deep learning-based statistical techniques can make predictions for novel uncharacterized materials and invent completely new materials [20]. Machine learning can express complex function mappings without the knowledge of structural features, and they form to be an effective estimation technique in the analysis of nonlinear behaviors. Input and output relationship of the system can be predicted through machine learning irrespective of the understanding of detailed mechanisms [9,21]. Previous studies have found that skyrmion is not limited to the magnets with DM interaction, such as B20, but widely exists in magnetic materials, thus requiring the assistance of high-throughput calculation [22–24]. However, there is no effective method to predict the accuracy of chiral crystal formation, and it is even more difficult to predict the coexistence of chiral geometries when multiple atoms are mixed [25,26]. For this kind of high dimensionality problem, the choice of features and classification schemes is crucial to achieving the system with high predictive performance [27–30]. Various logical models have been optimized to find a statistical technique to predict whether a substance is a skyrmion. In this work, machine learning algorithms, such as ensembles of trees, linear regression models, support vector machine (SVM), *k*-nearest neighbor (KNN), and naive Bayes, are used to analyze the dataset. Their abilities to classify materials were compared, and the model with the highest accuracy of 87.5% was obtained.

## Results and Discussion

**Fig. 1.**
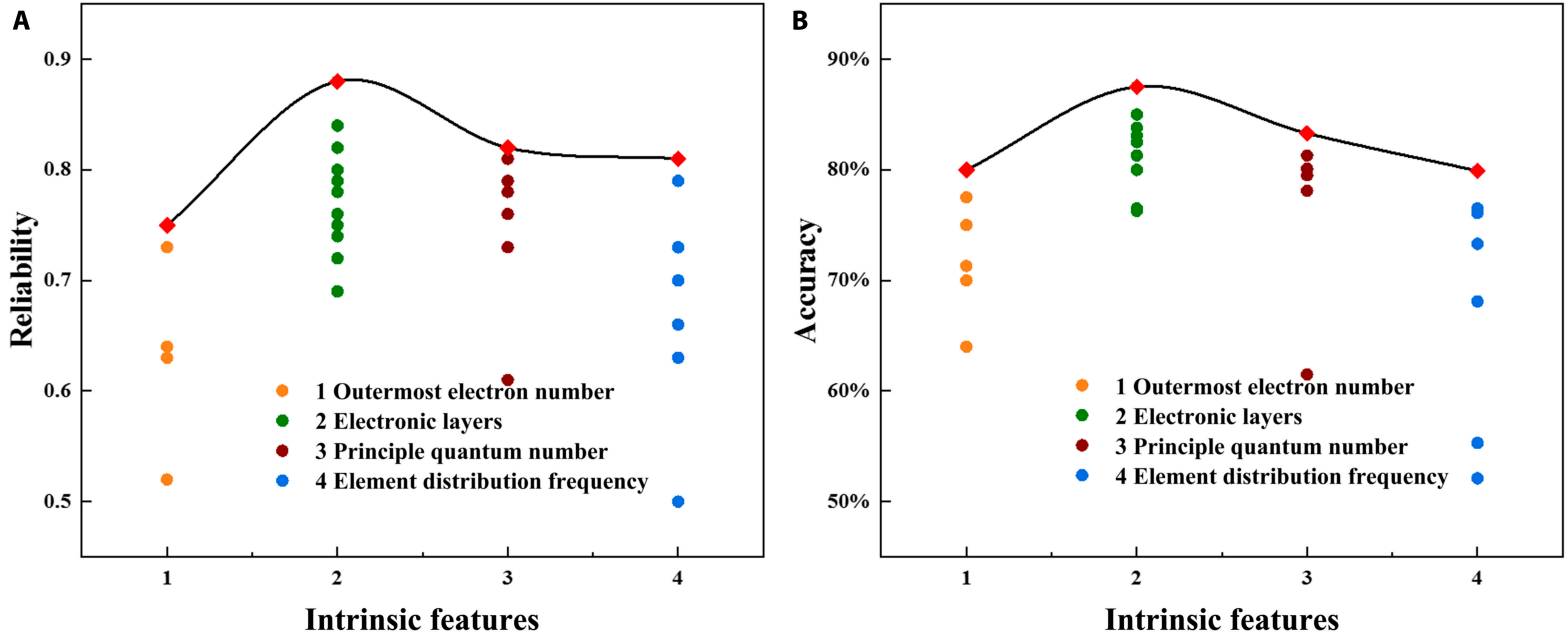
Reliability (A) and accuracy (B) of models under different intrinsic features, such as outermost electron number, electronic layers, principal quantum number, and element distribution frequency. Data points of the same color represent the results of different algorithms using the same classification.

Data science needs to find an appropriate classification method, especially when the amount of data is limited. The electronic layer, outermost electron number, principal quantum number, and element occurrence frequency, which have great influence on the material properties, were selected as intrinsic features. The difference between electronic layer classification and principal quantum number classification is that the former uses rare earth as an independent group based on the latter. From the accuracy and reliability of the models in Fig. 1, it can be seen that the classification based on the electronic layer works best. For models with poor performance, increasing the number of training sessions or providing more data in model creation may improve accuracy. However, the effect of the model is difficult to be stable, indicating that there is a serious overfitting at this time.

As shown in Table 1, the accuracy and reliability of different algorithms based on electronic layer classification are mostly above 76.3% and 0.72. Compared with other algorithms, the constraint relationship between predictors and target parameters in linear models may lead to lower prediction accuracy, such as logistic regression and linear discriminant. The performance of bagged trees, boosted trees, KNN, and SVM is introduced in detail below. The chemical composition of each material is represented by a linear combination of weighted contributions from these element classes. For example, Fe_3_Sn_2_ is described as 0.6 × label 3 + 0.4 × label 4. Labels refer to different electronic layers, which can be found in Table 2. Note that the rare earth elements with electronic layers of 6 are included as a separate group to make the model work better.

**Table 1.** Classification accuracy and reliability values for different proposed methods based on electronic layers.

Method	Accuracy	Reliability
RUS bagged trees	87.5%	0.89
Boosted trees	87.4%	0.86
Bagged trees	82.5%	0.82
Weighted KNN	81.3%	0.82
Cubic SVM	83.8%	0.80
Quadratic SVM	82.5%	0.79
Coarse Gaussian SVM	80.0%	0.79
Linear SVM	76.3%	0.78
Squad SVM	82.5%	0.76
Cubic KNN	82.5%	0.76
Subspace discriminant	81.3%	0.75
Logistic regression	83.8%	0.74
Medium KNN	82.5%	0.74
Cosine KNN	81.3%	0.72
Neural network	77.2%	0.75
Linear discriminant	76.5%	0.72
Medium Gaussian SVM	85.0%	0.69
Fine Gaussian SVM	80.0%	0.65
Naive Bayes	53.7%	0.58

**Table 2.** The elements are divided into labels 1 to 6 according to the principal quantum number.

	Label 1	Label 2	Label 3	Label 4	Label 5	Label 6
Number of electron layers	2	3	4	5	6	6&RE
Elements	B, O, F	Si, Na, Al, Mg, S	Fe, Co, Mn, Ge, Cu, Se, Ga, Ni, Cr, As, Br, Sc, V	Sn, Sr, Cd, Te, Y, Mo, Pd, Te, I	Ta, Bi, Pt, Ba	La, Nd, Gd, Tb, Ho, Er, Yb

### Bagged trees

**Fig. 2.**
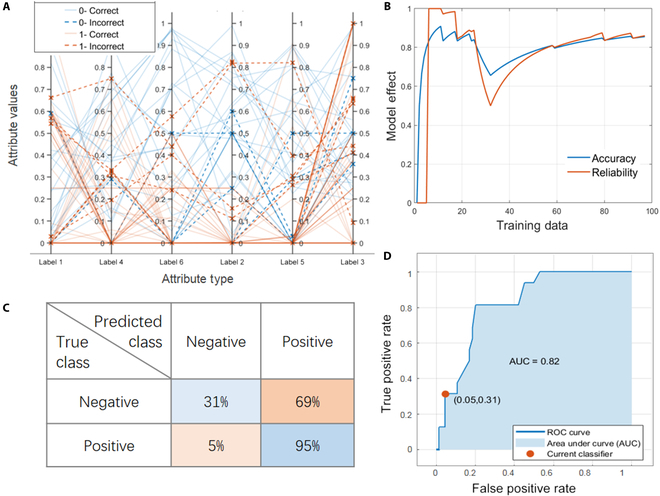
Model performance obtained after training bagged trees algorithm. (A) Parallel coordinator plot. (B) The accuracy and reliability of the model were calculated as a function of training data. (C) Confusion matrix. (D) ROC curve, which is used to evaluate its predictive ability.

We used the bootstrap method to create different samples from the original data and constructed the forest using the independent decision trees generated from each sample [31–34]. The results predicted from bagged trees can reduce overfitting and be more stable due to the differences between bootstrap samples. Compared with traditional decision trees, bagged trees is an integrated model that can solve the problems of poor stability and data fragmentation. As can be seen in Fig. 2A, parallel coordinate plot (PCP) was used to realize the visualization of high-dimensional multivariate data [35]. PCP shows the importance of each label in attribute type and the interrelation between labels. Each vertical line in this figure represents the class of electronic layer, and each sample is shown as a broken line running through all the vertical lines. As one can see, the lines on label 3 are more chaotic. This is because most materials contain elements in label 3, resulting in little influence of this label on the prediction. However, the same types of lines on labels 4 and 6 are concentrated, and the different types of lines are scattered, indicating that these labels are of great help in predicting the skyrmion category.

Figure 2B shows the changes in model accuracy and reliability with the increase of training data. When the training data are around 30, the model effect drops sharply, indicating that the newly added data have an adverse impact on the model. However, this part of data has been well learned with the increase in the number of iterations and the amount of data. The accuracy and reliability are also gradually improved and stabilized. Receiver operating characteristic (ROC) curve based on confusion matrix (Fig. 2C) was used to evaluate the predictive ability. Figure 2D shows the ROC curve obtained after the stabilization of the algorithm, where the abscissa is the false positive rate (FPR) and the ordinate is the true positive rate (TPR). The FPR only has an error of 5%, indicating that the probability of misjudging the material as skyrmion is very small. The accuracy of bagged trees classifier is 82.5%, and the area under ROC curve (AUC) is 0.82. AUC is a comprehensive measure of the effect of all possible classification thresholds and is intended to determine the degree of reliability or the ability to correctly predict categories. AUC ranged from 0.5 to 1, with the higher the better. Bagged trees algorithm relies on many decision trees rather than a single decision tree, which allows the model to leverage the insights of multiple models. However, the increased complexity and randomness of the computational process make bagged trees difficult to interpret. Appropriate classification of bagging during the analysis of material data can make it easier for computers to find correlations between components. Boosted trees and random undersampling (RUS) bagged trees described below are both variations of bagged trees.

### Boosted trees and RUS bagged trees

**Fig. 3.**
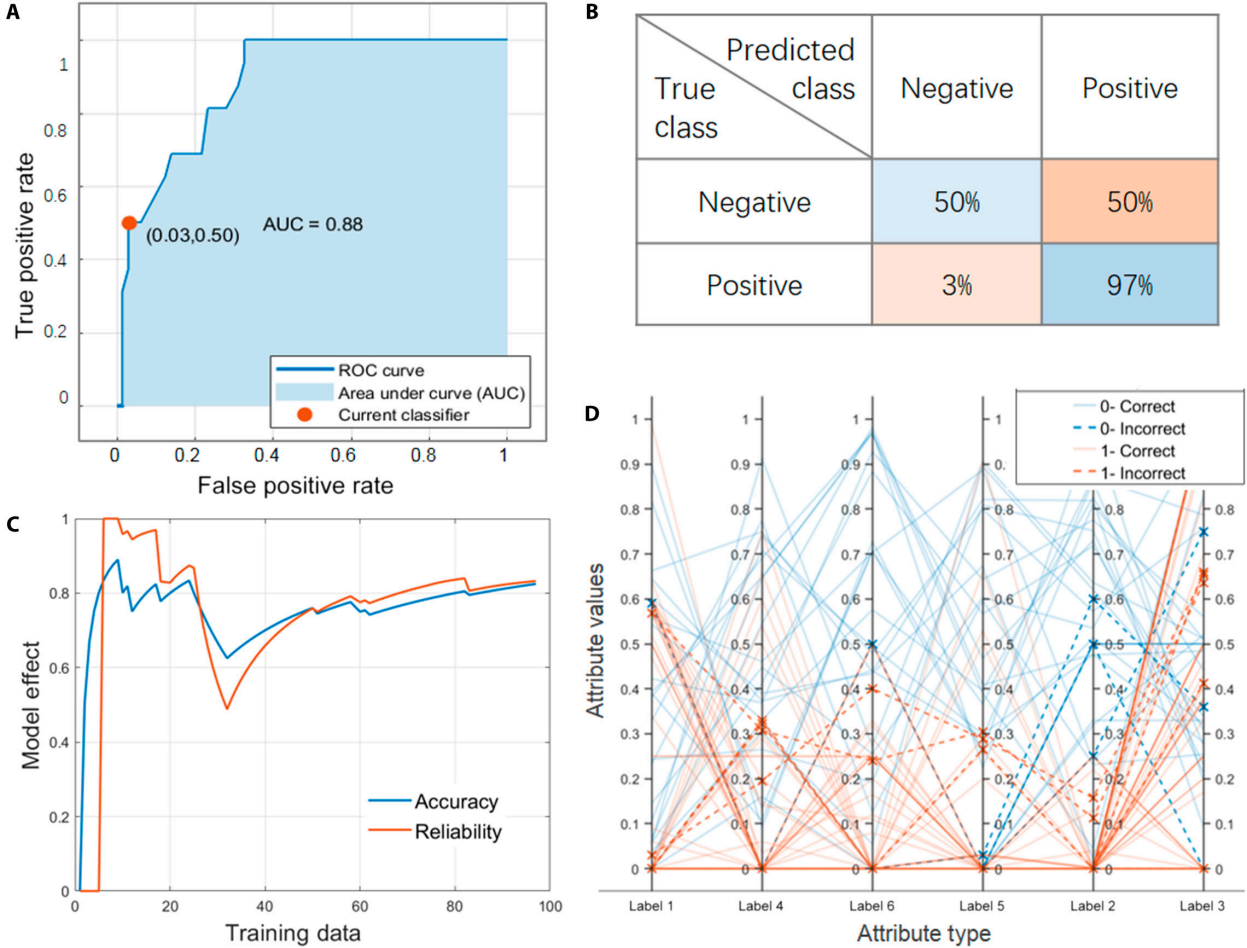
(A) ROC curve of boosted trees. (B) Confusion matrix of boosted trees. (C) Accuracy and reliability of RUS bagged trees vary with training data. (D) PCP represents the influence of different labels on classification of RUS bagged trees.

Boosted trees and RUS bagged trees are the most ideal models in this work with high accuracy and AUC value. The bootstrap samples of boosted trees are not randomly selected. When a decision tree is generated, boosted trees algorithm checks it against the entire sample and generates a new decision tree by minimizing the false predictions of all previous trees [32,36]. We use the adaptive boosting algorithm, and the accuracy of forest prediction is constantly improved with the sequential construction of decision trees. The AUC value in Fig. 3A is 0.88, which is higher than the result of bagged trees. Figure 3B evaluates the performance of the classifier by comparing positive (1) or negative (0) skyrmion data categories of the evaluation set with trained prediction categories. The TPR of this algorithm is 97%, which is very suitable for evaluating the performance of classification models. However, there are some defects in false negative prediction, which may be caused by insufficient interference terms in the data. Future improvements can be made by, for example, increasing the sample size to improve the accuracy of the predictions.

As shown in Fig. 3C, bagged trees with RUS has the accuracy and reliability of 87.5% and 0.89, respectively. The number of instances in the majority class was undersampled to solve the problem of class imbalance. It has been reported that the undersampling scheme based on heterogeneous consensus clustering has better prediction performance [37,38]. The importance of each label and how the labels relate to each other were analyzed according to PCP. The attribute value is obtained in the form of standard deviation of true or false skyrmion as depicted in Fig. 3D. The lines and colors on label 3 are mixed, indicating that this attribute is not helpful for the determination of skyrmion category. This is because most magnetic materials contain the elements in label 3, regardless of whether they are skyrmion materials. However, the same color polylines on label 4 to 6 axes are more concentrated, and different colors have certain spacing. Rare earth elements, in particular, have shown a key role in predicting material categories. Previous studies have also found that rare earths are beneficial to the formation of helical magnetic structures and adjustment of magnetic crystal anisotropy due to their strong spin-orbit coupling and high atomic magnetic moment [39–41]. For example, the substitution of Sc^3+^ induced the transformation of hexagonal ferrites from collinear to helical magnetic structures, and skyrmion domains were observed at room temperature without magnetic field. Therefore, rare earth was divided into a separate group for better modeling.

### Support vector machine

**Fig. 4.**
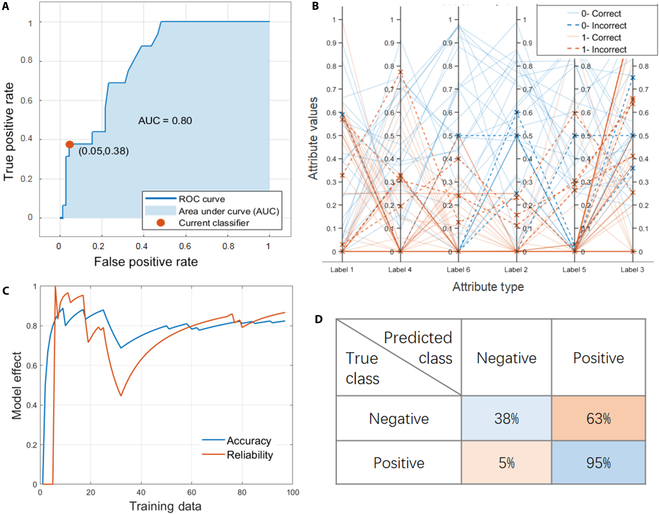
Model performance obtained after training cubic SVM. (A) ROC curve, used to represent TPR against the FPR at various thresholds. (B) Parallel coordinator plot. (C) Accuracy and reliability vary with training data. (D) Confusion matrix.

SVM is a machine learning algorithm, which can handle high-latitude data and adapt to the small dataset, and has also shown its applicability in this project. We use different kernel functions such as linear kernel, cubic kernel, quadratic kernel, and Gaussian kernel to ensure the nonlinear fitting ability of SVM model and reduce the complexity of vector inner product operation. However, it is difficult to adjust parameters and the training speed is slow. Since the model is not prone to overfitting, a better model can be obtained by increasing the training times while increasing the amount and dimension of data. It is found that the SVM with cubic kernel function has the highest accuracy. PCP in Fig. 4 shows that the elements in labels 2 and 6 have a great effect on the formation of skyrmion. The AUC value of cubic SVM is 0.80, which is slightly lower than the result of boosted trees, but still has a good effect. The poor false negative rate indicates that the model lacks understanding of the skyrmion material system. Seow and Ziegler [42] suggested a remedial measure to overcome the underprediction problem. It involves synthetically increasing the proportion of the high-value points using bootstrapping or oversampling so that the relative proportion of the high-value data points becomes large. The environmental engineering problem is at least 30 times larger than the skyrmion dataset. Therefore, experimental validations are still needed to test the underprediction problem and provide feedback for machine learning model improvement.

### Weighted KNN

**Fig. 5.**
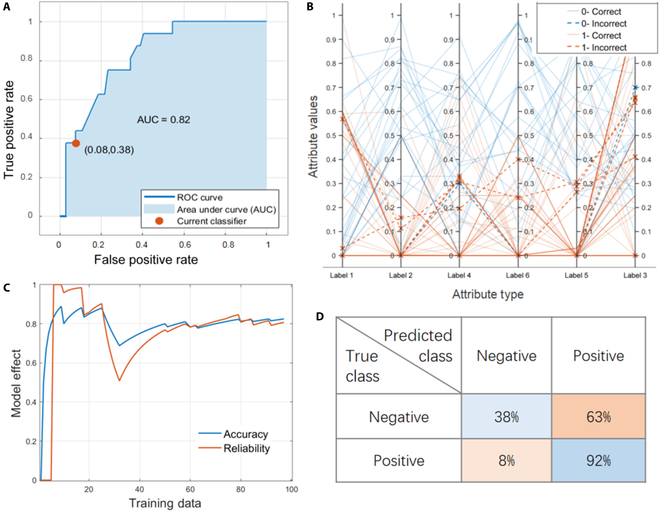
Model performance obtained after training weighted KNN. (A) ROC curve, used to represent TPR against the FPR at various thresholds. (B) Parallel coordinator plot. (C) Accuracy and reliability vary with training data. (D) Confusion matrix.

The dataset based on different weighted KNN methods was trained for magnetic domain prediction. Among the medium KNN, cosine KNN, cubic KNN, and weighted KNN algorithms, the last one is considered to be the best option. Other KNN algorithms have relatively low accuracy and weak stability, which are not suitable for machine learning with high-latitude data. As the simplest and most common machine learning method, KNN has a simple principle and better applicability after weighting. Weighted KNN is an improved algorithm on the basis of fine KNN, and its accuracy and reliability are optimized to 82.5% and 0.76, respectively. The PCP in Fig. 5 also shows that label 6 plays an important role in predicting skyrmion categories. Compared with SVM, weighted KNN has slightly lower accuracy, but higher reliability and practicability. It is because the AUC value of SVM is relatively low, which may be due to underfitting and strong randomness caused by insufficient data.

**Fig. 6.**
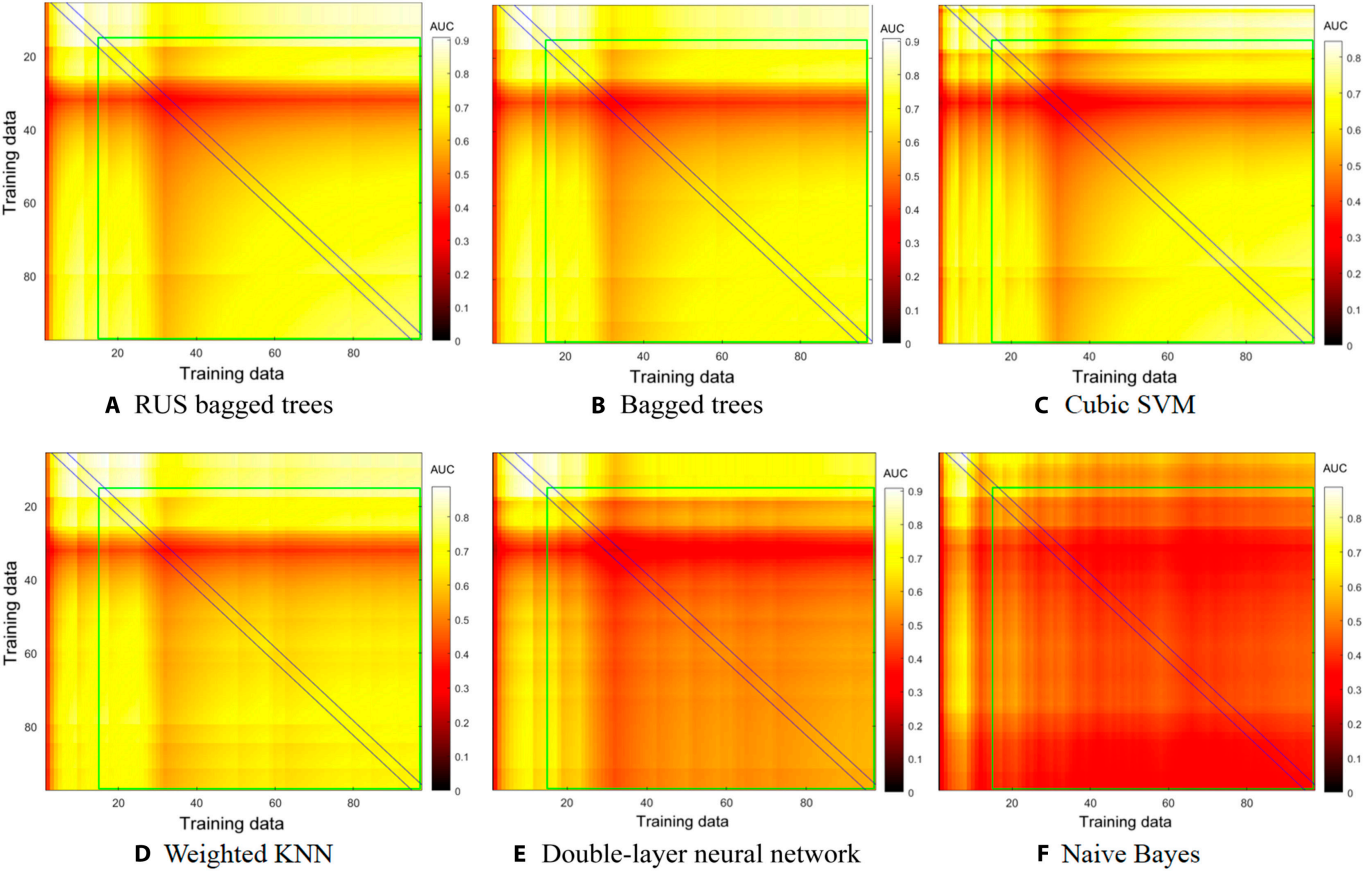
Hot spot map for different algorithms. (A) RUS bagged trees. (B) Bagged trees. (C) Cubic SVM. (D) Weighted KNN. (E) Double-layer neural network. (F) Naive Bayes. The area between the black lines shows the product of accuracy and reliability at the corresponding training dataset. The performance of models (A) to (F) gets worse progressively.

Figure 6 shows the hot spot maps of RUS bagged trees, bagged trees, cubic SVM, weighted KNN, double-layer neural network, and naive Bayes. As we know, there is a restrictive relationship between accuracy and reliability, that is, the higher the accuracy, the lower the reliability. Hot spot map was used to show the combined effect of accuracy and reliability with the number of training data. The lighter the color inside the green box, the better the prediction of the model (the training data outside the green box are too small to be referenced). The area between the black lines shows the product of accuracy and reliability in the corresponding training dataset. It is found that when the 25 to 32 sets of data are added to the training, the color of hot spot map suddenly became darker. That is, these data have a detrimental effect on the models, which is consistent with the results obtained above. This is mainly because these data contain a large number of new elements that were not included in the previous dataset, leading to improper adaptation of the model. However, the accuracy and reliability gradually recover with the increase of data size and training times, indicating that the model has learned this part of data. In contrast, neural network and naive Bayes model in Fig. 6F performed the worst. Such models cannot reduce the dimension of feature space and are insensitive to the strength of different features [43]. Although the uniform color expresses that the reliability of naive Bayes is stable, its accuracy is only about 53.7%, making it difficult to analyze high-dimensional data and find the hidden relationship between data. In the empirical analysis, the conventional deep neural network architectures combined with the weighted functions can improve the prediction performance [44,45].

**Fig. 7.**
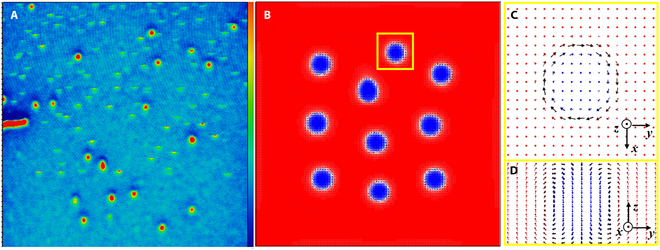
(A) Magnetic domain structure of LaBaMnO film in a magnetic field around 3,500 Oe and (B) corresponding simulation results. The in-plane and lateral magnetic moment distributions in the yellow boxes are shown in (C) and (D), respectively.

As can be seen from the confusion matrix in Figs. 2 to 5, our models have a high TPR and are suitable for prediction. Therefore, we can use the models to predict the new skyrmion material. The confidence that Sr(Fe_1−*x*_Sc*_x_*)_12_O_19_ contains skyrmion in the range of 0.05 to 0.3 is 79%, indicating that it is most likely to be skyrmion material. In addition, La_2/3_Ba_1/3_MnO_3_ alloy, which was not part of the dataset, was taken as a test case to verify the model trained by bagged trees. According to machine learning results under electron layer classification, the probability of the existence of skyrmion domain in LaBaMnO is more than 80%, which indicates that this material most likely contains skyrmion. This conclusion is verified by the micromagnetic and experimental results. The domain structure of LaBaMnO film prepared by pulsed laser deposition under 500 Oe magnetic field is shown in Fig. 7. The corresponding simulation results also show that LaBaMnO can form stable skyrmion domains. This simple, yet powerful, exercise demonstrates the predictive capabilities of trained machine learning models and can be used to rapidly predict the topological properties of previously unexplored alloy compositions.

## Conclusion

In this work, the skyrmion database is established on the foundations of machine learning. Machine learning algorithms such as logistic regression, SVM, decision trees, KNN, naive Bayes, and neural network were used to classify and analyze the data. It is found that the number of electronic layers is a suitable intrinsic feature for element classification, and more than 10 models can meet the application conditions. The RUS bagging trees had the best effect, with the accuracy and reliability of 87.5% and 0.89, respectively. The RUS bagging trees show the potential to build reliable machine learning models from small data. The PCPs show that rare earths in label 6, although not abundant in the materials, play an important role in the prediction of skyrmions. The trained machine learning was used to predict the magnetic domain of LaBaMnO, and the possibility of becoming skyrmion material was confirmed by micromagnetic simulation and experiment. Without complex techniques and advanced modalities, reliable results were obtained using only commonly accessible properties, which provide a basis and guidance for the field of auxiliary materials science and crystal design.

## Methods

**Fig. 8.**
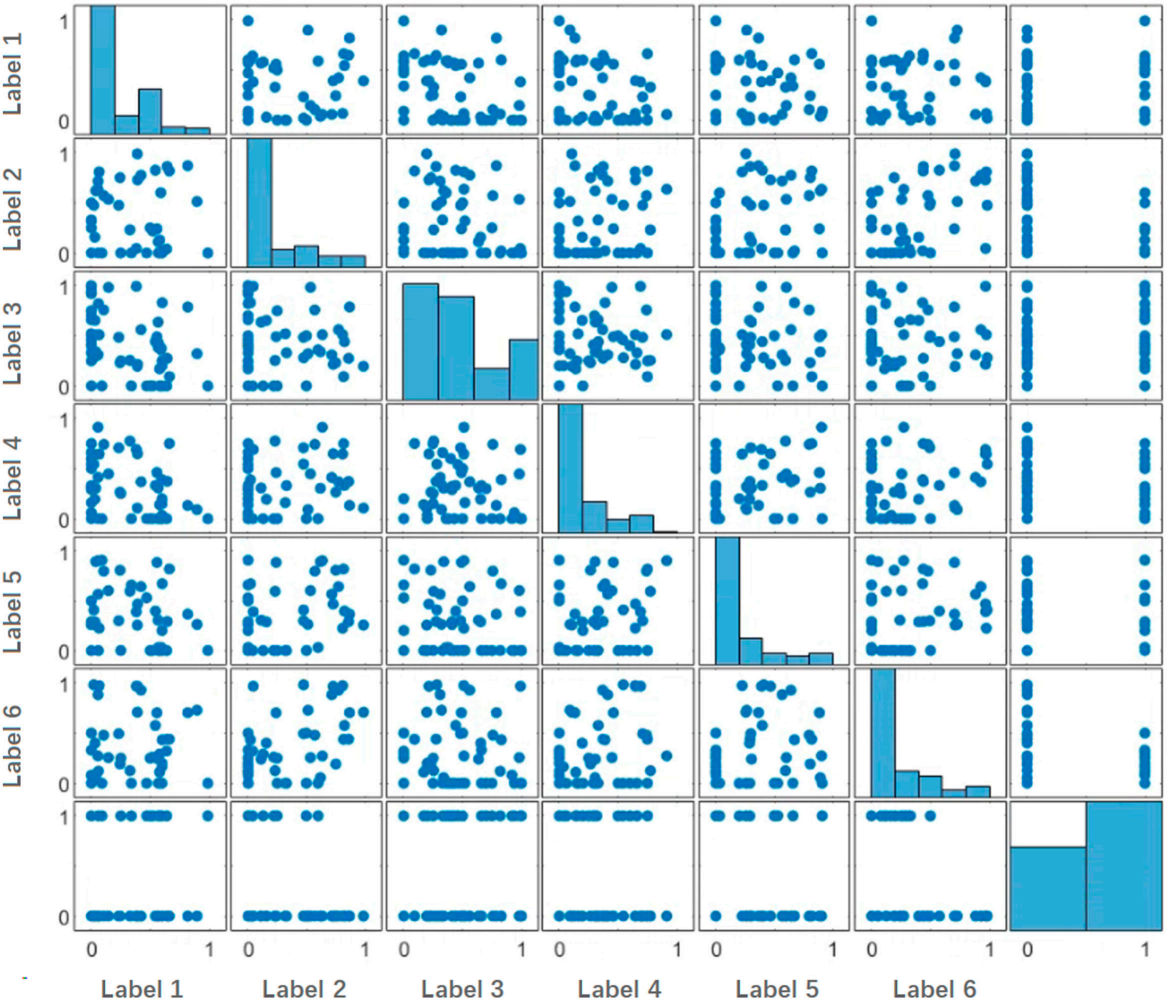
Data distribution matrix. The subplot in the *i*th row, *j*th column of the figure is a scatterplot of the *i*th column against the *j*th column. The subaxes along the diagonal are histogram plots of the corresponding classes.

Machine learning can be used in materials science to achieve pre-experimental prediction. In this work, a database of 196 materials, including 64 kinds of skyrmions, was obtained and analyzed according to different classification methods. More data about non-skyrmion could help prevent the occurrence of allergies. Electronic layers, outermost electron number, principal quantum number, and element occurrence frequency were selected as features and applied to classifier. Further probabilistic computation was performed to find out the chance of true or false skyrmion in the input dataset. Taking electron layer classification as an example, as shown in Fig. 8, we divided the elements involved in the materials into 6 labels. The subplot in *i*th row, *j*th column of the matrix is a scatterplot of the *i*th column against the *j*th column. For instance, row 1 and column 2 in this figure is a scatterplot with label 2 as abscissa and label 1 as ordinate. The subplots along the diagonal are replaced with histogram plots of the data in the corresponding column. The subplot in the first row, first column refers to the data distribution density of label 1, which can be used to investigate the effect of this label on the formation of skyrmion. The closer the value gets to 1, the more elements of this label are present in skyrmion materials. All data are represented by the subplot in row 7 and column 7, and the category of skyrmion is represented by 1 and 0.

**Fig. 9.**
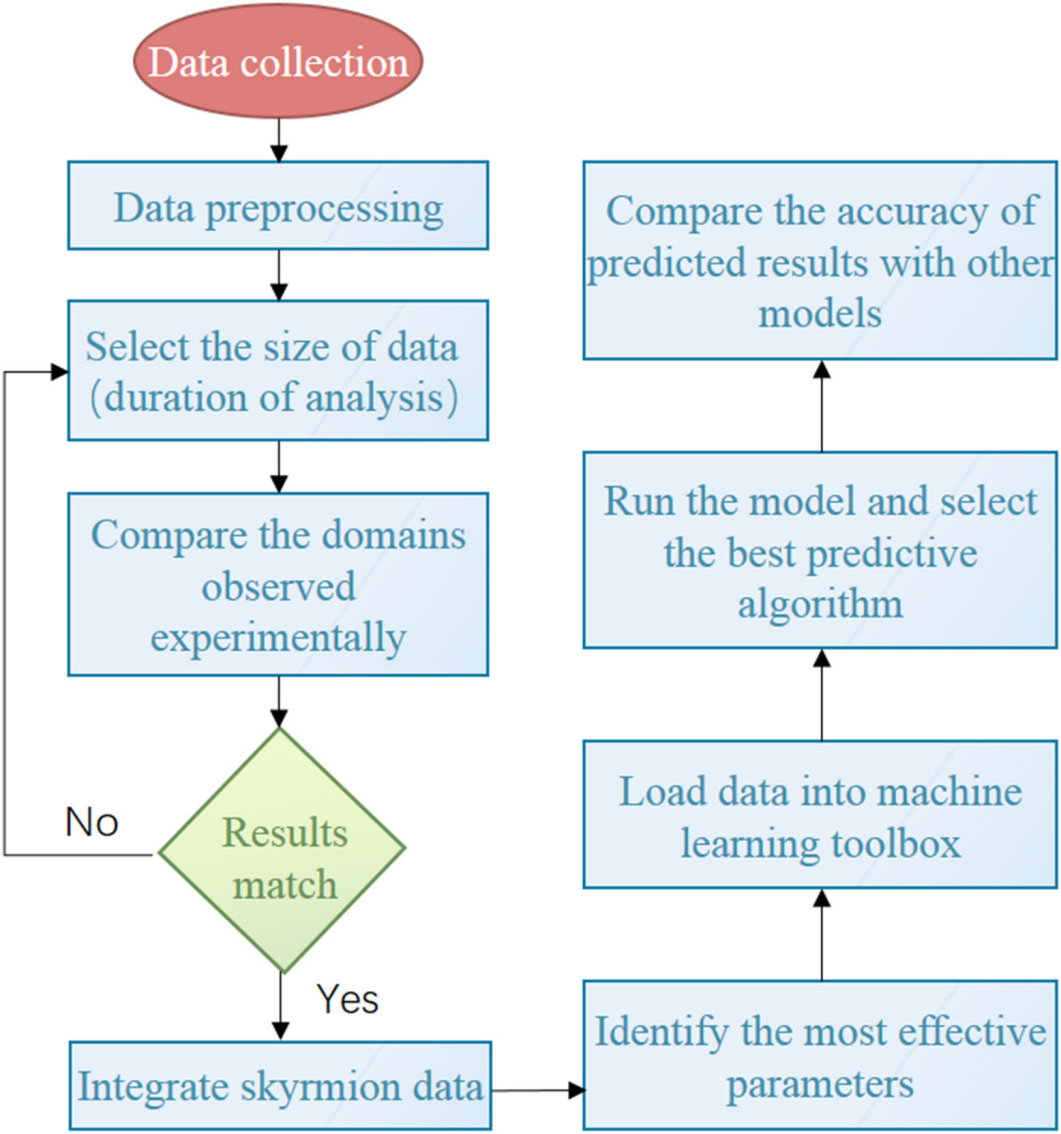
Schematic diagram of the process of data-driven strategy using machine learning models.

Figure 9 shows the procedure of data-driven strategy, and the different machine learning algorithms are listed in Table 3 [31,46,47]. Kernel functions are used to convert the input data from linearly nonseparable to linearly separable. The greater the separation, the better the performance. SVMs use linear, squad, quadratic, and cubic kernels to establish the relationship between the input descriptors and the presence or absence of skyrmion. In addition, the SVM methods of fine Gaussian, medium Gaussian, and coarse Gaussian are also involved [48]. KNN models can preprocess the raw data collected from the data logger, such as removing irrelevant and noisy data from the dataset to improve accuracy [49]. We also use 2 types of ensembles of trees, bagged trees, and boosted trees, which combine similar (or different) algorithms to provide more accurate predictions.

**Table 3.** Machine learning algorithms and corresponding subcategories used for skyrmion prediction in this work.

Algorithm	Subcategory
Linear regression models	Linear discriminant
	Logistic regression
Support vector machines (SVMs)	Linear SVM
	Squad SVM
	Quadratic SVM
	Cubic SVM
	Fine Gaussian SVM
	Medium Gaussian SVM
	Coarse Gaussian SVM
*K*-nearest neighbor (KNN)	Medium KNN
	Cosine KNN
	Cubic KNN
	Weighted KNN
Ensembles of trees	Bagged trees
	Boosted trees
	RUS bagged trees

## Data Availability

The authors confirm that the data supporting the findings of this study are available within the article.
